# A brassinosteroid functional analogue increases soybean drought resilience

**DOI:** 10.1038/s41598-022-15284-6

**Published:** 2022-07-04

**Authors:** Lucia Sandra Perez-Borroto, María Carla Guzzo, Gisella Posada, Andrea Natalia Peña Malavera, Atilio Pedro Castagnaro, Justo Lorenzo Gonzalez-Olmedo, Yamilet Coll-García, Esteban Mariano Pardo

**Affiliations:** 1 Plant Breeding, Wageningen University and Research, 6708 PB Wageningen, The Netherlands; 2 Instituto de Fisiología y Recursos Genéticos Vegetales Victorio S. Trippi - Unidad de Estudios Agropecuarios (IFRGV-UDEA, INTA-CONICET), Av. 11 de septiembre 4755, CP X5014MGO Córdoba, Argentina; 3 Instituto de Tecnología Agroindustrial del Noroeste Argentino (ITANOA), Estación Experimental Agroindustrial Obispo Colombres (EEAOC) /Consejo Nacional de Investigaciones Científicas Y Técnicas (CONICET), Las Talitas, Tucumán Argentina; 4 Centro de Bioplantas, Universidad de Ciego de Ávila “Máximo Gómez Báez”, Ciego de Ávila, Cuba; 5 Centro de Estudios de Productos Naturales, Facultad de Química, Universidad de La Habana, Havana, Cuba

**Keywords:** Plant physiology, Plant hormones, Brassinosteroid, Plant stress responses, Drought

## Abstract

Drought severely affects soybean productivity, challenging breeding/management strategies to increase crop resilience. Hormone-based biostimulants like brassinosteroids (BRs) modulate growth/defence trade-off, mitigating yield losses; yet, natural molecule's low stability challenges the development of cost-effective and long-lasting analogues. Here, we investigated for the first time the effects of BR functional analogue DI-31 in soybean physiology under drought by assessing changes in growth, photosynthesis, water relations, antioxidant metabolism, nodulation, and nitrogen homeostasis. Moreover, DI-31 application frequencies' effects on crop cycle and commercial cultivar yield stabilisation under drought were assessed. A single foliar application of DI-31 favoured plant drought tolerance, preventing reductions in canopy development and enhancing plant performance and water use since the early stages of stress. The analogue also increased the antioxidant response, favouring nitrogen homeostasis maintenance and attenuating the nodular senescence. Moreover, foliar applications of DI-31 every 21 days enhanced the absolute yield by ~ 9% and reduced drought-induced yield losses by ~ 7% in four commercial cultivars, increasing their drought tolerance efficiency by ~ 12%. These findings demonstrated the practical value of DI-31 as an environmentally friendly alternative for integrative soybean resilience management under drought.

## Introduction

Drought is the most prevalent abiotic stress in global agriculture^1^. Soybean (*Glycine max* (L.) Merrill), the most worldwide cropped legume, is strongly affected by water scarcity periods that cause reductions in photosynthesis and nitrogen (N) fixation, compromising grain quality and yield^2^. Strategies such as conventional/molecular breeding and cultural management practices^3^, like biostimulant applications^4^, have been implemented to mitigate drought effects. Biostimulants based on plant growth regulators such as brassinosteroids (BRs) integrated agricultural systems to optimise crops productivity, especially under unfavourable environments^4^.

BRs are involved in plant growth and development regulation and abiotic stress adaptation^5^. Its exogenous application or the genetic manipulation of their endogenous levels can alleviate the damage caused by drought^6^. However, BR's low stability in the field prevents large-scale application, being replaced by analogues with higher activity and average life^7^. Despite its high production fees, the most commonly used is 24-Epibrassinolide (EBL)^5^; thus, research of cheaper analogues with similar/higher activity constitutes a priority.

This research used the functional analogue DI-31, a synthetic spirostanic molecule, the active ingredient of the commercial formulation BIOBRAS 16, and characterised by a spiroketalic ring instead of the typical BR side-chain^8^. Applications of DI-31 improved the photosynthetic rate and yield of greenhouse-grown pepper ^9^ and endive plants^10^. Similarly, the BIOBRAS 16 prevented the negative effect of salinity in rice and lettuce^11,12^. In comparative studies with EBL, authors reported that foliar applications of BIOBRAS 16 in strawberries induced greater protection against *Colletotrichum acutatum*^13^. Meanwhile, in *Arabidopsis thaliana* (L.) Heynh plants submitted to drought, the DI-31, showed stronger and longer-term activity than EBL^14^.

However, no studies have yet reported the effects of DI-31 on legumes. Hence, we aimed to characterise, for the first time, the analogue action in soybean plants submitted to water deficit for potential drought resilience. Water scarcity interferes with plant growth, nutrient and water relations, photosynthesis and assimilates partitioning, ultimately causing substantial reductions in productivity^15^. In this context, we explore the DI-31 effects on plants’ performance, photosynthesis, biomass production and partitioning, canopy development, hydric status maintenance, enzymatic and non-enzymatic antioxidant responses, membrane stability and leaf pigments, osmolytes and sugars production. In legumes, environmental constraints, particularly drought, considerably affects nodulation and nitrogen fixation^16^. Therefore, we also assessed soybean nodulation and N homeostasis changes under drought and DI-31 treatments.

It is known that legumes tolerance/sensitivity to drought is variable, but in all cases, the final yield is drastically decreased^3^. Thus, plants' absolute yield and components were measured to determine the effect of DI-31 application frequencies throughout the soybean cycle and on the Drought Tolerance Efficiency (DTE) index.

## Results

### Effects in soybean physiology under drought

**Figure 1:**
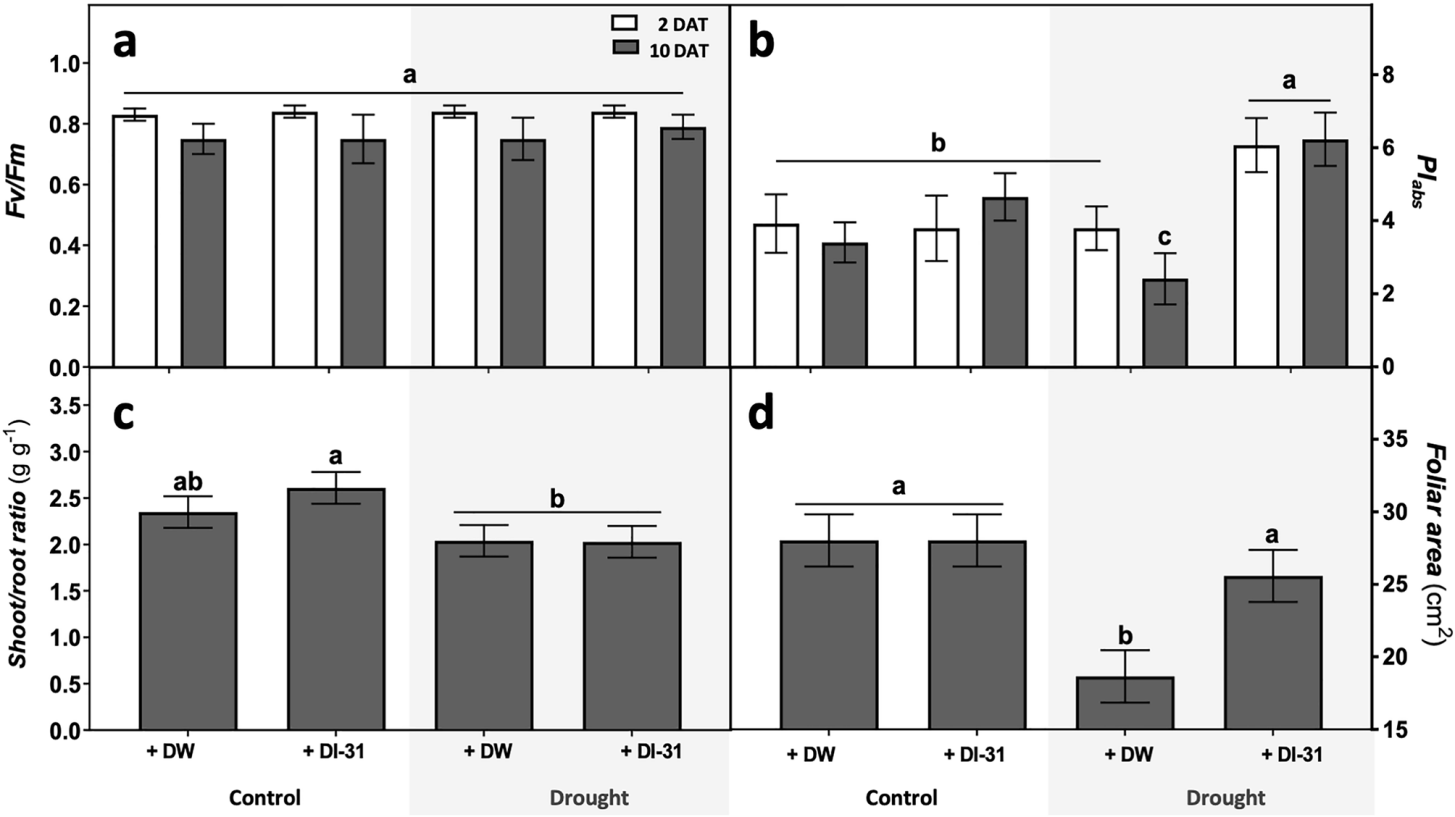
Effect of distilled water (DW) and DI-31 (2.23 µM) foliar applications in soybean growth and photosynthesis. Morphophysiological parameters such as (**a**) the maximum quantum yield of PSII (Fv/Fm), (**b**) plant performance index on absorption basis (PI_abs_), (**c**) shoot/root ratio and (**d**) foliar area were measured in *cv* Munasqa plants submitted to well-watered (Ψs = − 0.05 MPa) and drought (Ψs = − 0.65 MPa) conditions. Colour bars indicate parameters assessed after two (white) or ten days (grey) of stress and foliar treatments. Data are presented in means ± s.e.m of two independent experiments (n = 200). Different letters indicate significant differences (*P* ≤ 0.05) ANOVA with post hoc contrasts by Tukey's test.

To assess the overall effects of DI-31 application in soybean physiology under drought, we measured a wide range of indicators associated with photosynthesis, growth, water relations, stress-response, nodulation and nitrogen homeostasis. When analysing photosynthesis indicators measured after two and ten days of drought and DW (distilled water) or DI-31 treatments, plant Fv/Fm or maximum quantum yield of photosystem II (PSII) showed no differences among treatments and times (Fig. 1a). Regarding the performance index on an absorption basis (PI_abs_), a substantial decrease was detected in stressed plants treated with DW after ten days of drought, while the ones treated with DI-31 increased the PI_abs_ after two days (Fig. 1b).

After ten days of drought and DW/DI-31 applications, growth indicators like the shoot/root ratio considerably increased due to DI-31 in well-watered conditions (Fig. 1c), while the foliar area showed a ~ 34% fall only in drought-stressed plants (Fig. 1d). Complementary parameters like the root fresh/dry weights ratio, leaf area ratio (LAR), specific leaf area (SLA) and leaf weight ratio (LWR) can be found in Supplementary Fig. S1 online. Here, the root fresh/dry weights ratio remained unaltered (Fig. S1a). At the same time, LAR (Fig. S1b) and SLA (Fig. S1c) were increased due to DI-31 action under both well-watered and drought conditions. Regarding the LWR, a major reduction was found only in drought-stressed plants (Fig. S1d).

**Figure 2:**
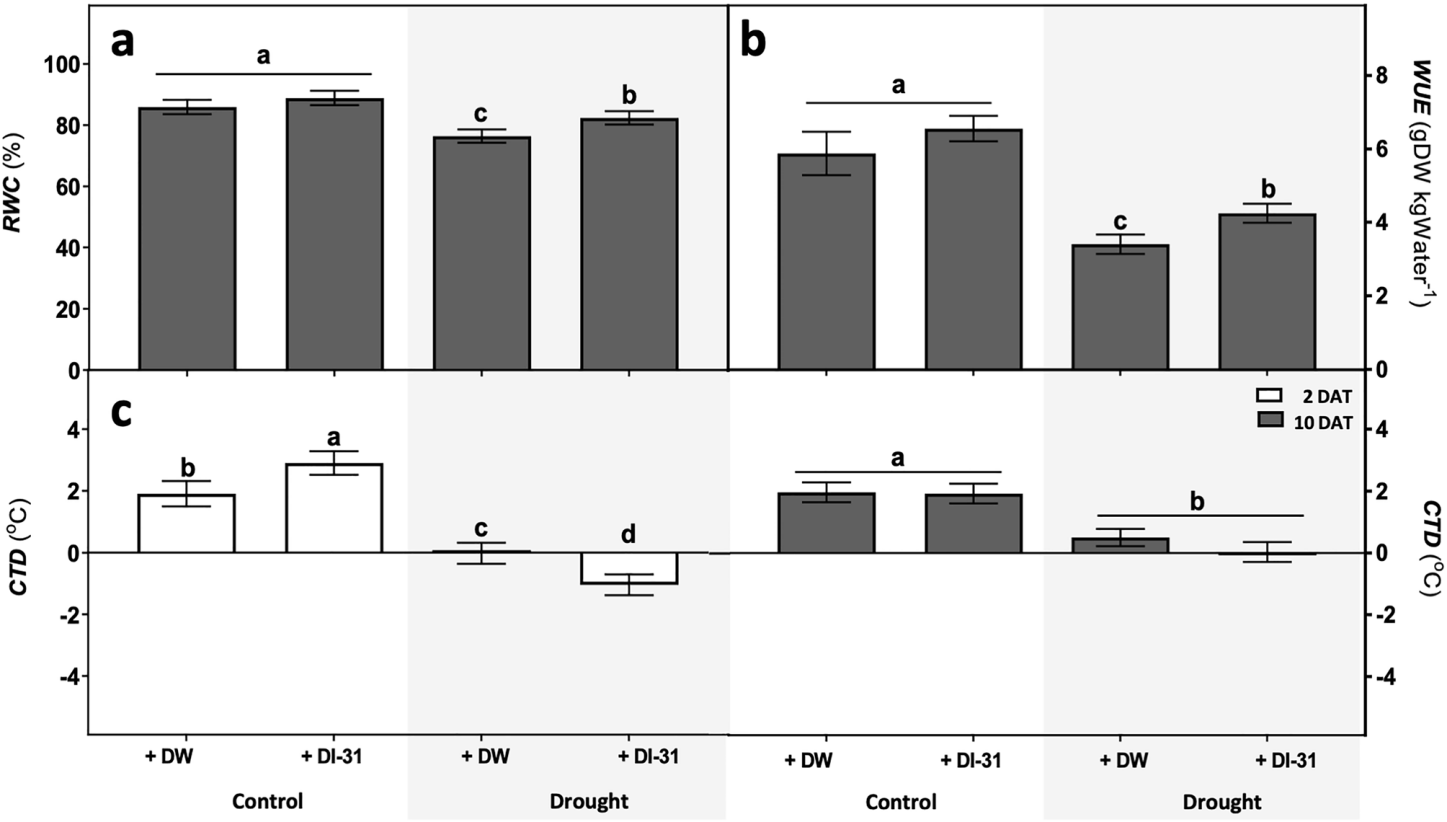
Effect of distilled water (DW) and DI-31 (2.23 µM) foliar applications in soybean water relations. Parameters such as (**a**) relative water content (RWC), (**b**) water use efficiency (WUE), and (**c**) canopy temperature depression (CTD) were measured in *cv* Munasqa plants submitted to well-watered (Ψs = − 0.05 MPa) and drought (Ψs = − 0.65 MPa) conditions. Colour bars indicate parameters assessed after two (white) or ten days (grey) of stress and foliar treatments. Data are presented in means ± s.e.m of two independent experiments (n = 200). Different letters indicate significant differences (*P* ≤ 0.05) ANOVA with post hoc contrasts by Tukey's test.

The DI-31 application modulated the water relations parameters under drought (Fig. 2). Here, plants submitted to mild water deficit exhibited a ~ 11% relative water content (RWC) reduction, while the stressed and treated with the analogue showed a ~ 7% decrease (Fig. 2a). The DI-31 also attenuated the water use efficiency (WUE) reduction under stress (Fig. 2b). Regarding the canopy temperature depression (CTD) (Fig. 2c), after two days of drought, well-watered plants treated with DI-31 exhibited the highest values, while under stress, those treated with the analogue showed the lowest CTD. Moreover, after ten days of mild water deficit, plants CTD only differed due to water availability treatments.

**Figure 3:**
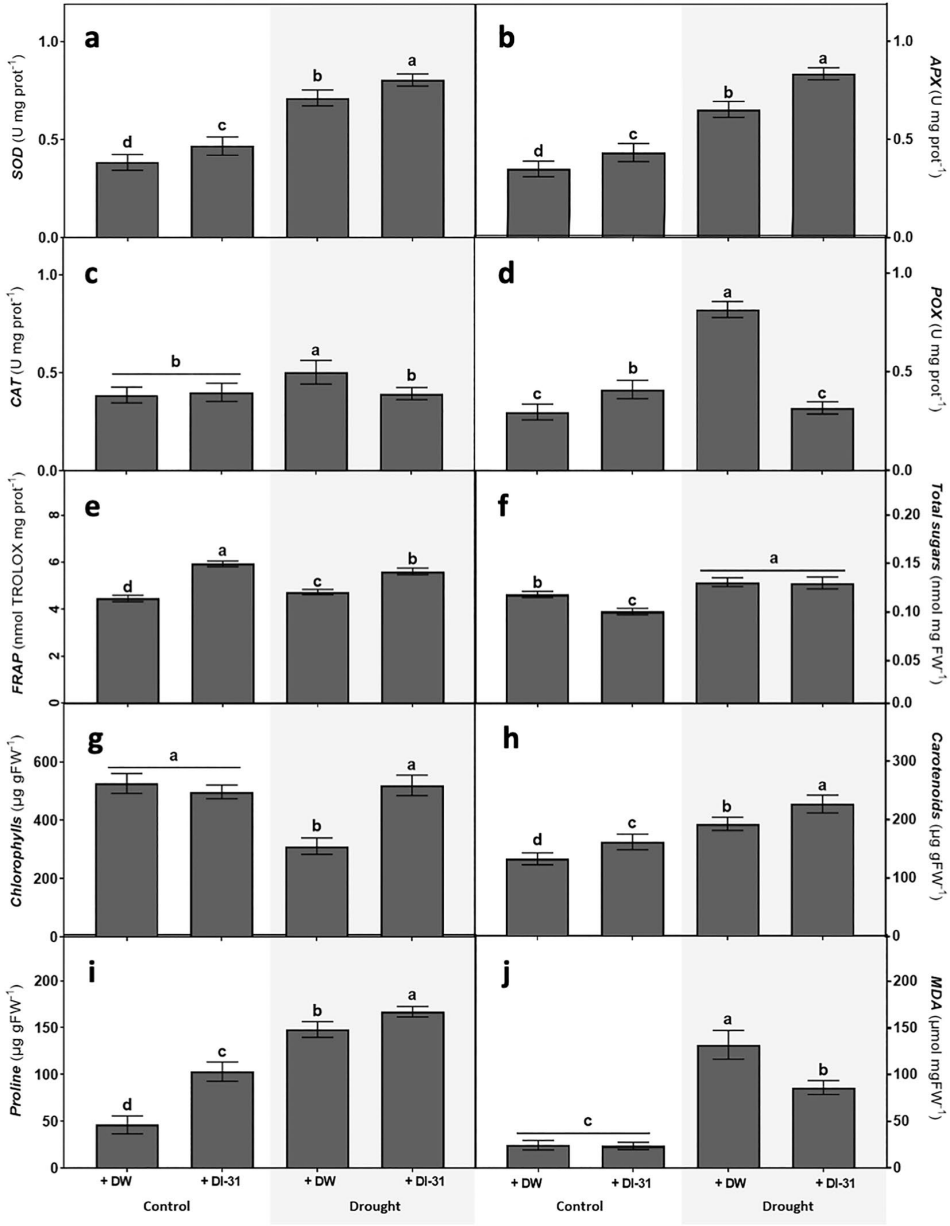
Effect of distilled water (DW) and DI-31 (2.23 µM) foliar applications in soybean stress response. Parameters such as (**a**) superoxide dismutase (SOD), (**b**) ascorbate peroxidase (APX), (**c**) catalase (CAT), (**d**) phenol peroxidase (POX), (**e**) ferric reducing ability potential (FRAP), (**f**) total sugars, (**g**) chlorophylls, (**h**) carotenoids, (**i**) proline and (**j**) malondialdehyde (MDA) content were measured in *cv* Munasqa plants submitted to well-watered (Ψs = − 0.05 MPa) and drought (Ψs = − 0.65 MPa) conditions for ten days. Data are presented in means ± s.e.m of two independent experiments (n = 120). Different letters indicate significant differences (*P* ≤ 0.05) ANOVA with post hoc contrasts by Tukey's test.

When analysing the DI-31 effects on stress response indicators, results showed that the analogue stimulates enzymatic and non-enzymatic antioxidants, precluding chlorophyll loss and malondialdehyde (MDA) accumulation (Fig. 3). Under well-watered conditions, the DI-31 application increased superoxide dismutase (SOD) (Fig. 3a) and ascorbate peroxidase (APX) activities (Fig. 3b). Moreover, drought triggered the activity of both enzymes; yet, the highest levels were determined in stressed plants treated with DI-31. In addition, an increment of phenol peroxidase (POX) activity was observed in well-watered plants sprayed with the analogue and in drought-stressed ones treated with DW (Fig. 3c). Regarding catalase (CAT) activity, an increase was only observed in drought-stressed plants (Fig. 3d). When observing the total non-enzymatic antioxidant capacity, a slight increase was observed in drought-stressed plants (Fig. 3e). In contrast, the ones treated with DI-31, under well-watered or drought conditions, exhibited the highest ferric reducing ability potential (FRAP) values. Furthermore, the content of total sugars showed a reduction in well-watered plants treated with DI-31 and a substantial increase in both stress treatments (Fig. 3f). Regarding the chlorophyll content (Fig. 3g), a reduction was detected only in the plants submitted to drought. The carotenoid (Fig. 3h) and proline (Fig. 3i) content were enhanced due to the DI-31 effect in both water availability conditions, where the drought-stressed ones exhibited the highest accumulation. As expected, drought increased the MDA content (Fig. 3j), especially in stressed plants treated with DW.

**Figure 4:**
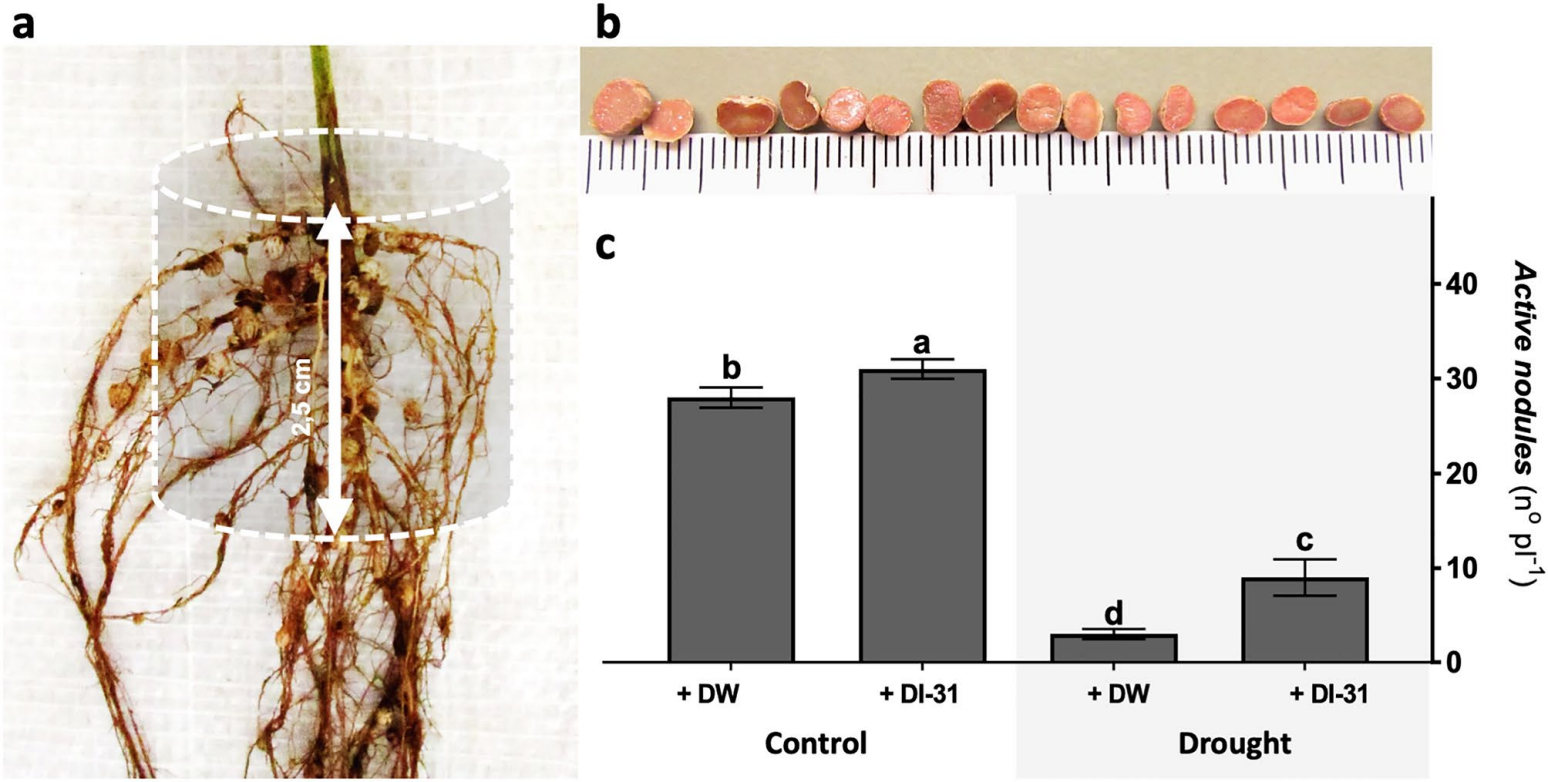
Effect of distilled water (DW) and DI-31 (2.23 µM) foliar applications in soybean nodulation. Nodules located in (**a**) the central axis of the primary root were collected, those with (**b**) light pink leghemoglobin colouration were considered active, then (**c**) the number of active nodules per plant was quantified in *cv* Munasqa plants submitted to well-watered (Ψs = − 0.05 MPa) and drought (Ψs = − 0.65 MPa) conditions for ten days. Data are presented in means ± s.e.m of two independent experiments (n = 120). Different letters indicate significant differences (*P* ≤ 0.05) ANOVA with post hoc contrasts by Tukey's test.

Regarding nodulation, a growth-promoting effect was observed in well-watered plants treated with DI-31, while in the stressed ones, the compound application produced a protective effect (Fig. 4). Ten days after a single foliar application of DI-31, the nodules located in Munasqa's imaginary root cylinder (Fig. 4a) exhibited substantial changes regarding its activity (Fig. 4b). Due to DI-31 action, the number of active nodules *per* plant increased by ~ 10% under well-watered conditions (Fig. 4c). Under drought, the DW-treated plants showed a ~ 57% reduction of active nodules that was lessened by ~ 26% due to DI-31 application.

**Figure 5:**
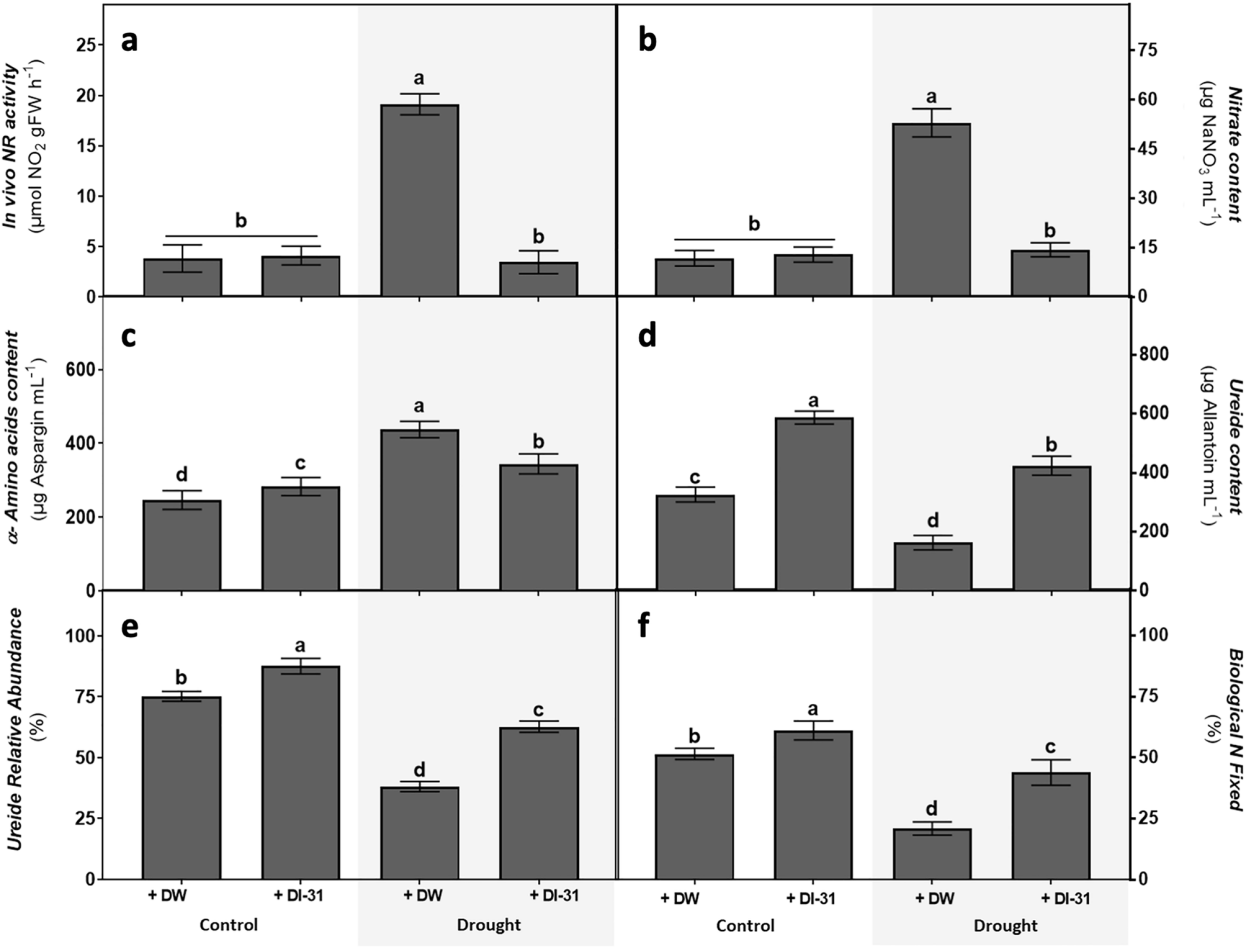
Effect of distilled water (DW) and DI-31 (2.23 µM) foliar applications in soybean N homeostasis. Parameters such as (**a**) in vivo nitrate reductase (NR) activity, (**b**) nitrate content, (**c**) α-amino acids content, (**d**) ureide content, (**e**) ureide relative abundance and (**f**) biological N fixed were measured in *cv* Munasqa plants submitted to well-watered (Ψs = − 0.05 MPa) and drought (Ψs = − 0.65 MPa) conditions for ten days. Data are presented in means ± s.e.m of two independent experiments (n = 120). Different letters indicate significant differences (*P* ≤ 0.05) ANOVA with post hoc contrasts by Tukey's test.

The nitrogen homeostasis parameters showed major alterations in the plants treated with the DI-31 (Fig. 5). The in vivo nitrate reductase (NR) activity (Fig. 5a) and the nitrate content (Fig. 5b) showed substantial increases only in plants submitted to drought treatment. In comparison with control plants, all treatments showed an increase in the content of α-amino acids (Fig. 5c), especially the DW-treated plants submitted to drought. An increase in biological N fixation parameters was detected in well-watered plants treated with DI-31, which showed an increase in ureide content (Fig. 5d); moreover, ureides relative abundance (Fig. 5e) and the percentage of biological N fixed (Fig. 5f) also increased by ~ 15% and ~ 16%, respectively. In contrast, plants submitted to drought and DW treatments reduced these parameters, considerably precluded by the DI-31 application.

The phenotypic variability observed in well-watered and drought-stressed plants treated with DW or DI-31 was corroborated by PCA analysis performed for all 28 parameters and subsets of data grouped by biological processes, and a double gradient heatmap. The corresponding methods, graphs and results can be found as Supplementary File S1 online.

### Application frequency effects in yield and yield components

**Figure 6:**
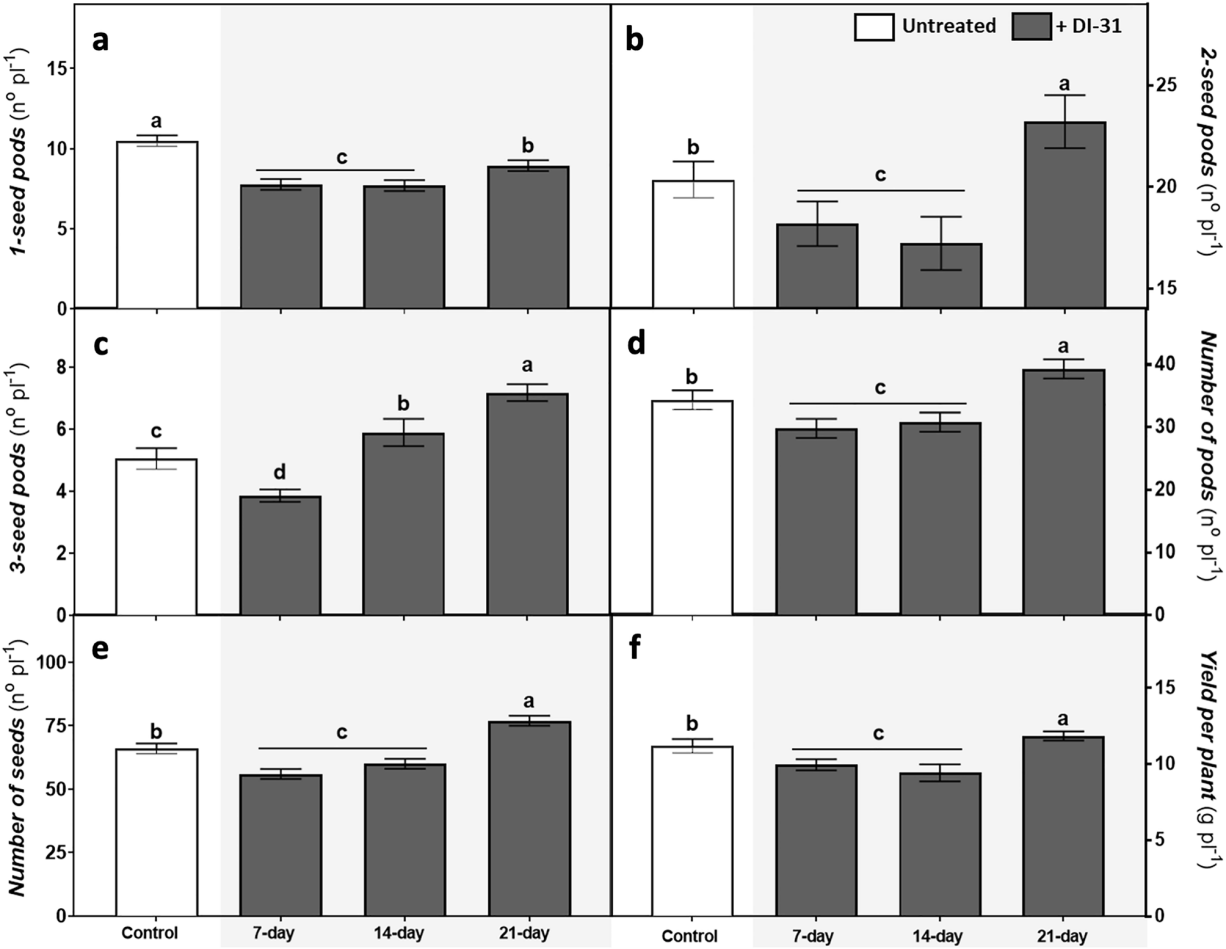
Effect of DI-31 (2.23 µM) foliar applications frequencies in soybean absolute yield and yield components. Parameters such as (**a**) number of pods with one seed *per* plant, (**b**) two seeds *per* plant, (**c**) three seeds *per* plant, (**d**) total pods *per* plant, (**e**) total seeds *per* plant and (**f**) absolute yield *per* plant were measured in *cv* Munasqa plants sprayed with DI-31 (2.23 µM; 1 mg/L) every 7, 14 or 21 days. Colour bars indicate control plants (white) or DI-31 treated ones (grey). Data are presented in means ± s.e.m of one independent experiment (n = 100). Different letters indicate significant differences (P ≤ 0.05) ANOVA with post hoc contrasts by Tukey's test.

To assess whether the DI-31 application frequency influences plant yield and yield components, the effects of three different frequencies were analysed in Munasqa plants (Fig. 6). Here, results showed that foliar treatments with DI-31 every seven days reduced the number of pods with one and two seeds by ~ 26% and ~ 11% (Fig. 6a,b), the number of pods with three seeds in ~ 24% (Fig. 6c), the total number of pods *per* plant in ~ 13% (Fig. 6d) and the number of seeds and absolute yield *per* plant in ~ 15% and ~ 11%, respectively (Fig. 6e,f). When analysing the plants treated with DI-31 every 14 days, similar overall effects were observed, except in the number of pods with three seeds *per* plant, which increased by ~ 17% due to the analogue action. The DI-31 foliar application every 21 days, compared to controls, decreased the number of pods with one seed *per* plant to a lesser extent (~ 14%), while increased in ~ 15% and ~ 42% the number of pods with two and three seeds *per* plant, the total pods and seeds *per* plant in ~ 14% and ~ 17%, also enhancing the absolute yield in ~ 6%.

### Yield-maintenance under drought

Based on the application frequency experiment results, the DI-31 effect on the number of seeds and absolute yield *per* plant of commercial cultivars was evaluated in Munasqa, NS8282, TJ2049 and DM5958 varieties submitted to drought in V_3_ and R_5_ stages (Table 1). Results showed that DI-31 applications every 21 days increased the number of seeds and the absolute yield *per* plant of all cultivars grown under optimal irrigation or submitted to drought at the V_3_ stage. Here, under well-watered conditions, the number of seeds and absolute yield *per* plant increased ~ 5% and ~ 6% in Munasqa, ~ 6% and ~ 8% in TJ2049 and NS8282, while in DM5958 cultivar augmented by ~ 11% and ~ 12%, respectively. The number of seeds and the absolute yield *per* plant were only reduced by the water deficit imposed at the R_5_ stage. When analysing drought/control ratios of R_5_-stressed cultivars, a major yield fall was observed (see Supplementary Fig. S2 online). Here, the drought reduced the cultivars number of seeds and absolute yield *per* plant by ~ 26% in Munasqa, ~ 53% in TJ2049, ~ 33% in NS8282 and ~ 43% in DM5958. These reductions were lessened due to DI-31 action in all the evaluated cultivars. Here, Munasqa exhibited a ~ 10% overall fall, while TJ2049 showed a ~ 42%, NS8282 a ~ 21% and DM5958 a ~ 32%. Additionally, under water deficit in R_5_, the DI-31 application increased the DTE by ~ 16% in Munasqa, ~ 12% in NS8282, and ~ 10% in TJ2049 and DM5958 cultivars (Table 2).

**Table 1:** Number of seeds and absolute yield *per* plant of four commercial soybean cultivars foliarly sprayed with distilled water or DI-31 every 21 days and submitted to a 10-day drought period at V_3_ or R_5_ stages.

Genotype	Treatment	No. seeds (n^o^ pl^−^^1^)		Yield (g pl^−^^1^)	
Munasqa	Control	68.6	E	11.2	F
	Control + DI31	72.3	D	11.9	E
	Stress-V_3_	69.3	E	11.3	F
	Stress-V_3_ + DI-31	71.7	D	11.8	E
	Stress-R_5_	50.8	F	8.3	I
	Stress-R_5_ + DI-31	61.2	E	10.1	G
TJ2049	Control	80.7	B	14.1	B
	Control + DI31	85.8	A	15.2	A
	Stress-V_3_	80.5	B	14.5	B
	Stress-V_3_ + DI-31	84.2	A	14.9	A
	Stress-R_5_	38.5	H	6.7	K
	Stress-R_5_ + DI-31	46.5	G	8.2	I
NS8282	Control	74.5	C	11.4	F
	Control + DI31	79.2	B	12.3	D
	Stress-V_3_	73.2	C	11.2	F
	Stress-V_3_ + DI-31	77.8	B	12.1	E
	Stress-R_5_	49.9	F	7.6	J
	Stress-R_5_ + DI-31	58.4	E	9.0	H
DM5958	Control	75.7	C	12.0	E
	Control + DI31	83.7	A	13.4	C
	Stress-V_3_	76.5	C	12.1	E
	Stress-V_3_ + DI-31	78.7	B	12.6	D
	Stress-R_5_	43.9	G	6.9	K
	Stress-R_5_ + DI-31	51.3	F	8.2	I
s.e.m		3.29		0.38	

Average values followed by the same uppercase letter do not differ statistically according to ANOVA with post hoc contrasts by Tukey's test (*P* ≤ 0.05).

**Table 2:** Drought Tolerance Efficiency (DTE) of four commercial soybean cultivars foliarly sprayed with distilled water or DI-31 every 21 days and submitted to a 10–day drought period at the R_5_ critical stage.

Genotype	DTE (%)	
	DW	DI-31
Munasqa	74	90
TJ2049	48	58
NS8282	67	79
DM5958	58	68

DTE calculations were based on the absolute yield *per* plant data.

## Discussion

Yield stabilisation and enhanced stress response are two main frameworks to identify drought tolerance traits in crops, including soybean^17^. The first considers yield variations in terms of growth and water relation features, while the other is associated with early stress-sensing and response traits. These two frameworks were considered when discussing the DI-31 applicability to mitigate the detrimental effects of water scarcity in soybean physiology.

Several legume studies reported unfavourable drought effects on biomass production and source/sink relations, reducing shoot and root development^3^. As growth promoters, BRs regulate plant development even under adverse environments^6^, modulating morphogenesis^18^ and assimilate production/translocation^19^. Our results demonstrated that a single foliar application of DI-31 protected Munasqa growth during ten days of drought, stabilising the foliar area development and biomass production and partitioning.

When analysing photosynthesis parameters such as the Fv/Fm, affected by drought progression in soybeans^20^, no changes were observed in Munasqa plants. This genotype is particularly drought-tolerant^21^; thus, under moderate water deficit, we did not expect reductions in the proportion of light absorbed by PSII chlorophyll and used in the photochemical processes^22^. Another indicator of photosynthetic fitness was the plant performance index on an absorption basis (PI_abs_). The PI_abs_ hosts three independent parameters that cumulatively quantify the total functionality of the electron flux through the PSII^23^. This indicator provides a valuable tool for evaluating plant performance under stress in terms of photosynthetic energy conservation^24^, thereby plant vitality^25^. We found that DI-31 application increased Munasqa PI_abs_ under drought, indicating an improvement in plant energy conservation since the early stages of stress. These results suggest the potential action of DI-31 as a growth stabiliser under drought.

Growth maintenance under drought also requires successful water management that involves the regulation of plant stomatal conductance, transpiration rate, RWC and WUE^3^. BRs influenced plant-water relations under normal and drought environments^26^, regulating the tissues hydric status and the relation between the water-consumed and the biomass produced^27^. Accordingly, our findings indicated that DI-31, applied at the stress onset, lessened the RWC and WUE drops induced by drought in Munasqa, favouring the hydric status maintenance and the biomass conversion with lower water cost. BRs also regulate stomatal movement, and therefore, the level of taken water *vs* transpired^18^. Here, the DI-31 regulated plant CTD, increasing foliar cooling under well-watered conditions and canopy heating under water deficit, especially at early stages of stress. CTD indicates how much leaves transpired and is considered a surrogate trait for the stomatal conductance^28^. Thus, we considered that the DI-31 might affect plant stomatal aperture/closure, increasing water-saving responses during drought periods. In previous studies, we corroborated that the analogue induces stomatal closure in a dose-responsive manner in Arabidopsis and, compared to EBL and ABA, the closure level fluctuated between partial and complete^14^. These results agreed with the ones detected in drought-stressed Munasqas treated with DI-31, where warmer canopies with CTD negative or near zero were observed together with higher RWC and WUE levels. These findings strengthen the DI-31 applicability to modulate water loss by transpiration, precluding major biomass penalties under drought.

Drought tolerance is a complex trait that encompasses growth stabilisation, water relations and several stress-sensing/response mechanisms^29^. In a physiological approach, drought sensing and response comprise, among others, reactive oxygen species (ROS) generation, detection and control via enzymatic and non-enzymatic paths^29^ that BRs can regulate to prevent ROS-induced injuries^30^. When analysing the DI-31 effects on Munasqa antioxidant response, our findings confirmed that the analogue, independently of the water availability, favoured the enzymatic scavenging of superoxide radicals (O_2_^−^) and hydrogen peroxide (H_2_O_2_). Moreover, the compound increased plants total non-enzymatic antioxidant capacity, the content of photoprotective pigments such as carotenoids and osmolytes like proline. These effects explain the attenuated chlorophyll loss and MDA accumulation detected in drought-stressed plants treated with the analogue.

Up to this point, we reported DI-31 effects in soybean growth, water relations and stress response regulation under well-watered conditions and drought. In agreement, several authors reported the beneficial effects of natural BRs and analogue molecules in crops development and tolerance/resistance to abiotic/biotic stresses^31^. However, few studies addressed the BRs effects on legumes nodulation and N homeostasis. In other crops, authors reported that foliar application of EBL considerably enhanced the activity of N-assimilation enzymes in tomato^32^ and pepper^33^. At the same time, Wang et al.^34^ suggest that the transcriptional factor BZR1, a BR's positive regulator, possibly plays a critical role in N-starvation response in tomatoes. Also, Cheng et al.^35^ reported that optimum levels of 24-epicastasterone regulated soybean growth and macronutrients homeostasis.

The N is a crucial constituent of proteins, nucleic acids and chlorophyll, playing a pivotal role in plants growth, development and productivity^36^. Legumes present high N demand but are often grown on soils with insufficient amounts of nitrate or ammonia forms^37^. As a result, the BNF or biological fixation of atmospheric dinitrogen (N_2_) is an efficient mechanism to increase N nutrition^38^. In legumes, the BNF occurs in the nodule, a specialised root organ resulting from plant-bacteria interactions^39^. Environmental stresses like water scarcity severely disrupt nodules developmental cycle^40^. Drought induces ROS and reactive N species (RNS) accumulation, Lb oxidation and Nitrogenase complex inactivation, leading to a fast senescence^41^, which can be easily monitored through the number of active nodules in roots. In this context, our findings corroborate the occurrence of drought-induced senescence and demonstrate the DI-31 protective effect.

In legumes, two major mechanisms regulate N homeostasis: the assimilative nitrate reduction and the BNF^3,42^. In the nitrate reduction path, the NR enzyme converts nitrate (NO_3_^−^) into nitrite (NO_2_^−^), subsequently transformed into ammonia (NH_3_), assimilable ammonium (NH_4_^+^) and finally α-amino acids, mainly asparagine^42^. In BNF, the NH_4_^+^ is converted into ureides, allantoin and allantoic acid, synthesised in the nodules and transported to the leaves through the xylem^43^. Here, different effects were found when analysing the DI-31 action in plant N homeostasis. In well-watered plants, the BNF parameters were increased, and these results agreed with the effects observed in nodulation and total sugar content in leaves.

The BNF maintenance requires the exchange of metabolites, as in any symbiotic relation; thus, plants transport large amounts of single and complex sugars into the nodules in exchange for ureides^44^. Thus, the decrease in total sugar concentration observed in well-watered plant leaves could be explained by the increase in BNF. In the meantime, drought-stressed plants increased NR activity, nitrate and α-amino acids contents, and reduced the leaf ureide content, relative abundance, and the percentage of N biologically fixed. Our findings, also in agreement with the nodulation results, indicated an increase in the assimilative nitrate reduction path in response to the BNF reduction. These drought-induced effects were lessened by DI-31 application, suggesting that the analogue favoured the N fixation drought tolerance (NFDT). However, it is unclear if BNF parameters like the ureide accumulation in leaves are associated with tolerant or susceptible responses to low water availability. Previous studies in drought-sensitive soybeans reported contradictory results. Vadez et al.^45^ described a decrease in leaf ureide content and Nitrogenase complex activity in response to water scarcity, while King et al.^43^ correlated the BNF decrease with the ureide accumulation in leaves. A subsequent study reported a lack of drought tolerance in soybean cultivars with low ureide content in leaves and increased concentration in roots^46^.

Meanwhile, Charlson et al.^47^ informed that, under stress, direct inhibition of BNF is triggered by the ureides accumulation in the nodule; therefore, if proper transport from the nodules to leaves is guaranteed, the BNF inhibition could be prevented, at least temporarily. The ureide concentration per se does not constitute an unequivocal indicator of NFDT in soybean^48^; thus, it must be assessed in diverse plant tissues or together with other BNF parameters. Nevertheless, we consider the occurrence of DI-31-plant-nodule interactions that positively modulated nodulation and N homeostasis and lessened the drought-induced nodular senescence and BNF inhibition.

Considering the findings discussed so far and in agreement with the PCA results and double gradient heatmap (see Supplementary File 1 online), we determined that the DI-31 modulates plant development and defensive mechanism, favouring growth, nodulation and N homeostasis under water availability conditions, and triggering water relations control and antioxidant metabolism in response to drought. Moreover, we consider that the DI-31 application might have an accumulative, and therefore, long-term action on soybean yield beyond the short and middle-term effects observed. These pleiotropic properties offer exciting potentialities for enhancing soybean productivity under favourable conditions and lessening yield losses under water-limited environments.

In this regard, extensive testing showed that exogenous applications of BRs substantially increased yield in several crops; yet, the increments can vary depending on the growth stage, mode and frequency of application^49^. Hence, to prevent any potential inhibitory effect, we first assessed the optimal application frequency of DI-31 throughout the Munasqa cycle. Here, results demonstrate that the analogue can induce defence-inductive or growth-promoting effects depending on the spraying frequency. Foliar treatments with DI-31 every 7 and 14 days exerted a growth inhibitory action, probably due to the analogue's defence-inductive properties. Conversely, applications every 21 days, a total of four from V_3_ to R_6_ stages, increased the absolute plant yield. Based on these findings, we preliminary confirmed the long-term action of DI-31, with a substantial impact on plant yield enhancement. Moreover, the growth-promoting maintenance observed in the plants treated every 21 days with DI-31 suggests that the compound might exert early and late effects, first triggering the defence-induction mechanism and, later, the growth-promoting one. Interestingly, the early effect (the defence induction) increased in response to drought; thus, the analogue might co-regulate plant defensive responses with other stress hormones. However, further research is needed to confirm if and how the DI-31 interacts with other stress hormones and which particular up/downstream regulatory elements are involved.

Meanwhile, to further analyse the DI-31 effect in soybean yield stabilisation under drought, the analogue action in Munasqa, NS8282, TJ2049 and DM5958 cultivars was evaluated using the 21-day application frequency. Here, water deficits were applied in the V_3_ early vegetative stage and the drought-sensitive R_5_ stage^21^. As expected, in well-irrigated plants, the DI-31 application increased the total number of seeds and absolute yield *per* plant by ~ 7% and ~ 9%, respectively. Several studies reported yield increments due to foliar applications of BRs in wheat, rice, maise, tobacco, sugar beet, cotton, rapeseed, tomato and potato^50^. Regarding the yield increase, our findings reinforced the DI-3 BRs-like activity and agreed with the compound growth-promoting effect observed in well-watered plants. BRs elicit diverse physiological responses that ameliorate the drought-derived impact on yield, and its application during early stress stages or moderate stress levels could preclude yield losses^50^. This could explain the absolute yield increments observed in DI-31-treated plants submitted to drought in the vegetative stage. Here, the parameters evaluated confirmed the drought occurrence; yet, it is possible to consider that the stress earliness and/or duration favoured the plant's recovery. The analogue action considerably enhanced this process; thus, we must consider its practical use to improve soybeans early-stress recovery.

Regarding the drought imposition in the R_5_ stage, as expected, all the cultivars showed yield reductions that fluctuated according to the drought-susceptibility of each variety (~ 48% in susceptible and ~ 30% in the tolerant ones). These findings confirmed the effectiveness of the stress imposed and corroborated that the reproductive stage, especially the grain-filling phase, is soybeans most drought-sensitive period. Interestingly, the yield reductions caused by drought treatment were lessened due to the DI-31 action (~ 11% and ~ 14% in susceptible and tolerant cultivars, respectively). In agreement with our results, several authors reported that BRs, like EBL, 28-homobrassinolide and brassinolide, reduced yield losses in several legumes like lentil, pea, mungbean, cowpea and soybean submitted to drought^50,51^. Previously, we discussed the DI-31 potential dual-action in soybean physiology as a BRs-like growth promoter and a defence-inductor enhancer. Here, we must highlight the analogue effect in yield stabilisation under drought and its practical importance in crop management strategies. Soybean cultivars submitted to DI-31 and water scarcity treatments showed increased plant Drought Tolerance Efficiency (DTE). However, the DTE increases observed differ a ~ 6% among the most drought-susceptible and tolerant cultivars. Hence, we consider that the analogue effect on drought-resilience enhancement might be proportional to the intrinsic drought tolerance of each variety.

Overall, our findings strengthen the practical value of DI-31 as a cost-effective and environmentally friendly alternative for modulating pivotal drought-resilience mechanisms like biomass production stabilisation, N homeostasis and plant productivity. Thus, its potential use in agriculture represents a sustainable alternative for alleviating drought-derived impacts on soybean production, contributing to integrative crop management amidst climate change threats.

## Methods

### Plant material and growth conditions

All experiments were conducted in greenhouse conditions at the Estación Experimental Agroindustrial Obispo Colombres (EEAOC), Las Talitas, Tucumán, Argentina (S26°50', W65°12'), and were performed following relevant guidelines and regulations. Seeds of Munasqa, NS8282, TJ2049 and DM5958 commercial cultivars were provided by the EEAOC Germplasm Bank with the corresponding permissions and used during experiments. Plants were grown in 4 L pots (diameter: 18 cm, height: 21 cm) filled with commercial substrate Grow Mix MULTIPRO (Terrafertil S.A., Argentina). Topsoil was covered with perlite to minimise water evaporation. Before sowing, seeds were inoculated with *Bradyrhizobium japonicum* E109 strain to guarantee maximum plant performance. Four seeds *per* pot were placed to ensure germination. At the V_1_ stage, open leaf at the unifoliate node^52^, one plant *per* pot was left. All experiments were performed in greenhouse conditions using plants in the V_3_ stage, second open trefoil, or the R_5_ stage, beans beginning to develop at one of the four uppermost nodes with a wholly unrolled leaf^52^. Plants grew under a 12-h photoperiod, 30 °C (± 3 °C) of average environmental temperature, ~ 90.0% relative humidity and photosynthetically active radiation of 648.37 μmol m^−2^ s^−1^. Pots were weekly rearranged to minimise possible environmental effects.

### Irrigation management

According to Pereira-Irujo et al.^53^, the substrate volumetric water content (VWC) *per* pot and the amount of water added daily to reach the desired VWC were estimated. Subsequently, the relationship between VWC and water potential (Ψ_w_) was determined according to Richards^54^. All pots were watered to a 22% VWC corresponding to a Ψ_w_ of − 0.05 MPa until the onset of drought. Stress imposition was performed according to Pardo et al.^21^ phenotyping protocol, where plants were submitted to mild water deficit at 14% VWC corresponding to a Ψs of − 0.65 MPa. The Ψ_w_ corresponding to water deficit was reached in a 1–2 days interval. Plant relative water content (RWC) was monitored throughout the water shortage period to ensure stress occurrence^55^. Pots were daily watered and weighed to quantify the amount of water evaporated from the substrate.

### Chemicals

The DI-31 was produced in the CEPN Synthesis Laboratory (Faculty of Chemistry, Havana University of Cuba). The TROLOX (6-hydroxy-2,5,7,8-tetramethylchromane-2-carboxylic acid) and the L-proline were obtained from Sigma-Aldrich (USA). The stock solution of DI-31 (22.3 mM) was prepared in 50% (v/v) ethanol, and the working solutions were prepared by diluting with distilled water (DW) and used immediately after.

### DI-31 effect in soybean physiology under drought

The DI-31 action in soybean *cv* Munasqa under drought was assessed in two independent experiments by evaluating photosynthesis, growth, water relations, stress response, nodulation, and nitrogen homeostasis parameters. Plants in the V_3_ phenological stage were sprayed with distilled water (DW) or DI-31 (2.23 µM; 1 mg/L) and submitted to ten days of water deficit. The following treatments were defined: (i) well-irrigated plants + DW, (ii) well-irrigated plants + DI-31, (iii) stressed plants + DW and (iv) stressed plants + DI-31. The DW and DI-31 were sprayed to drip point on the entire foliar region at the onset of stress. After two days of drought, photosynthesis and canopy temperature were non-destructively measured in 10 plants *per* treatment to assess changes at early stages of stress and BRs application. After ten days of water deficit, 30 whole plants *per* treatment were sampled (total of 120 plants), and different morphophysiological indicators were evaluated. Half of the collected plants, 15 *per* treatment, were used to assess growth and water relations parameters, together with nodulation. Leaf samples from the remaining plants were immediately ground in liquid nitrogen, stored at − 70 °C, and further used to determine stress response and nitrogen homeostasis indicators.

#### Photosynthesis

The Fv/Fm or maximum quantum yield of photosystem II (PSII) and the performance index on an absorption basis (PI_abs_) were assessed using a Pocket-PEA fluorometer (Plant Efficiency Analyser, Hansatech Instruments Ltd., King's Lynn, Norfolk, UK) as described by Strasser et al.^24^. Before measurements, shuttered leaf clips were adapted to darkness for 30 min to guarantee the total oxidation of the reaction centres. Next, a single strong 1-s light pulse (3500 μmol m^−2^ s^−1^) was applied in the 650 nm spectrum band.

#### Growth

The foliar area was non-destructively assessed using ImageJ Software (v.1.52)^56^. Fresh, turgor and dry weights were determined and used to calculate the shoot/root ratio. Fresh weights were quantified immediately after collecting the plants, whereas dry weights were measured after drying the samples for five days at 70 °C. Leaf area ratio (LAR), specific leaf area (SLA) and leaf weight ratio (LWR) were calculated according to Lin^57^.

#### Water relations

The RWC was measured according to Barrs et al.^55^. The canopy temperature, recorded with a dual laser infrared thermometer (HT-817), was used to calculate the canopy temperature depression (CTD)^58^. The water use efficiency (WUE), defined as the ratio between the above-ground biomass and the water consumed, was calculated according to Halsema et al.^59^.

#### Stress-response

A uniform enzymatic extraction^60^ was performed. The activities of superoxide dismutase (SOD, EC 1.15.1.1)^61^, catalase (CAT, EC 1.11.1.6)^62^, ascorbate (APX, EC 1.11.1.11)^63^ and phenol peroxidase (POX, EC 1.11.1.7)^64^ were assessed together with the total protein content^65^. The total non-enzymatic antioxidant capacity was evaluated with the Ferric Reducing Ability Potential (FRAP) assay^66^ and expressed as TROLOX equivalent in μmol *per* mg. Total chlorophylls^67^ and carotenoids^68^ were quantified. Proline content was measured through the ninhydrin assay modified by Guzzo et al.^69^, and the proline values were related to the L-proline calibration curve. The lipid oxidation by MDA accumulation was estimated through the thiobarbituric acid-reactive-substances (TBARS) assay modified by Guzzo et al.^69^. Based on the TBARS protocol, the total sugars content (sucrose, glucose and fructose) was estimated by subtracting the sugar absorbance maximum at 440 nm^70^.

#### Nodulation

All nodules located in the central axis of the primary root (2.5 cm diameter and length) were collected and cut to visualise their activity status according to the leghemoglobin (Lb) colouration^71^. The number of light pink nodules, considered mature and active, was quantified *per* plant.

#### Nitrogen homeostasis

The in vivo activity of nitrate reductase enzyme (NR, EC 1.7.1.1-3)^72^, nitrate^73^, α-amino acids^74^ and ureide content^75^ were quantified. Then, the ureide relative abundance^76^ and the percentage of biological N fixed^77^ were calculated.

### Effect of DI-31 application frequency in yield and yield components

The DI-31 optimal frequency of application throughout the Munasqa life cycle was assessed in an independent experiment. The following treatments were defined: (i) untreated plants as controls and plants foliarly sprayed with DI-31 (2.23 µM; 1 mg/L) every (ii) 7, (iii) 14 or (iv) 21 days. Plants were submitted to the different treatments from the V_3_ stage until physiological maturity. After harvest, yield components such as pods with one, two or three seeds, total pods and seeds *per* plant were quantified in 25 plants *per* treatment (100 total plants). Next, seeds were oven-dried at 70 °C for 48 h, and the absolute yield, in terms of total seed weight *per* plant, was calculated.

### DI-31 effect in yield-maintenance under drought

The DI-31 effect in yield was assessed in one independent experiment using Munasqa, NS8282, TJ2049 and DM5958 commercial cultivars. All genotypes were submitted to well-watered and drought treatments at V_3_ and R_5_ stages. The following treatments were defined: (i) well-watered plants (as controls), (ii) well-watered plants + D1-31, (iii) V_3_ drought-stressed plants, (iv) V_3_ drought-stressed plants + D1-31, (v) R_5_ drought-stressed plants and (vi) R_5_ drought-stressed plants + D1-31. The DI-31 was sprayed to drip point on the entire foliar region every 21 days starting from V_3_ until R_6_ stage, pods containing full-size green beans at one of the four uppermost nodes with a completely unrolled leaf^52^. The water deficit was maintained for ten days. At physiological maturity, 30 plants *per* treatment and genotype (total 720 plants) were manually harvested, and grains were oven-dried at 70 °C for 48 h. Subsequently, the absolute yield was quantified, and the genotypes Drought Tolerance Efficiency (DTE) index was estimated according to Fischer et al.^78^ formula.

### Statistical analysis

Data were analysed using InfoStat software^79^. Statistical analyses for the yield-maintenance experiments were performed over the raw data, and results from R_5_ experiments were expressed as the ln of the ratio stressed/control. The data were analysed using ANOVA and Tukey's test. Means were considered significantly different at P ≤ 0.05 and presented ± s.e.m.

Additional multivariate analyses were conducted using the data corresponding to the "*DI-31 effect in soybean physiology under drought*" experiments; methods, graphs and results can be found as Supplementary File S1 online.

### Ethics approval

The seeds of Munasqa, NS8282, TJ2049 and DM5958 commercial cultivars were provided by the EEAOC Germplasm Bank with the corresponding permissions for its use during this research. All the experiments conducted during this research were performed following relevant guidelines and regulations of the IUCN Policy Statement.

## Supplementary Information

Supplementary Information 1.

Supplementary Information 2.

Supplementary Information 3.

## Data Availability

The datasets generated during the current research are not publicly available due to confidentiality agreement; but are available from the corresponding author on reasonable request. Correspondence and requests for materials should be addressed to E.M.P.
