# Supplementary material for: A brassinosteroid functional analogue increases soybean drought resilience
Source: Sci Rep. 2022 Jul 4;12:11294. doi: 10.1038/s41598-022-15284-6 (PMC9253120; doi:10.1038/s41598-022-15284-6)
Supplement: Supplementary file 1 — Supplementary Information 1. [file 41598_2022_15284_MOESM1_ESM.pdf]

Manuscript: A brassinosteroid functional analogue increases soybean drought resilience

Supplementary Fig. S1

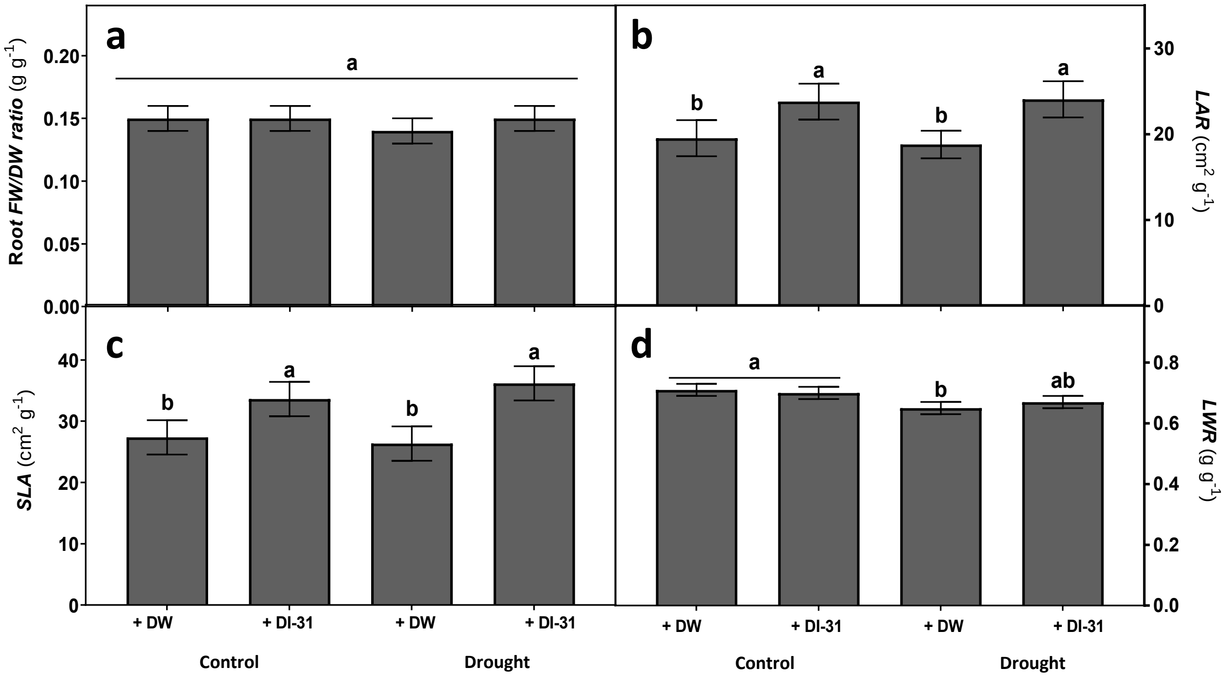

Fig. S1 Effect of distilled water (DW) and DI-31 (2.23 µM) foliar applications in soybean growth. Morphophysiological parameters such as (**a**) root fresh (FW)/dry weights (DW) ratio, (**b**) leaf area ratio (LAR), (**c**) specific leaf area (SLA) and (**d**) leaf weight ratio (LWR) were measured in *cv* Munasqa plants submitted to well-watered (Ψs = -0.05 MPa) and drought (Ψs= -0.65 MPa) conditions for ten days. Data are presented in means ± SE of two independent experiments (n=120). Different letters indicate significant differences ($P \leq 0.05$) ANOVA with *post hoc* contrasts by Tukey's test.
