# Supplementary material for: A brassinosteroid functional analogue increases soybean drought resilience
Source: Sci Rep. 2022 Jul 4;12:11294. doi: 10.1038/s41598-022-15284-6 (PMC9253120; doi:10.1038/s41598-022-15284-6)
Supplement: Supplementary file 2 — Supplementary Information 2. [file 41598_2022_15284_MOESM2_ESM.pdf]

Manuscript: A brassinosteroid functional analogue increases soybean drought resilience

Supplementary Fig. S2

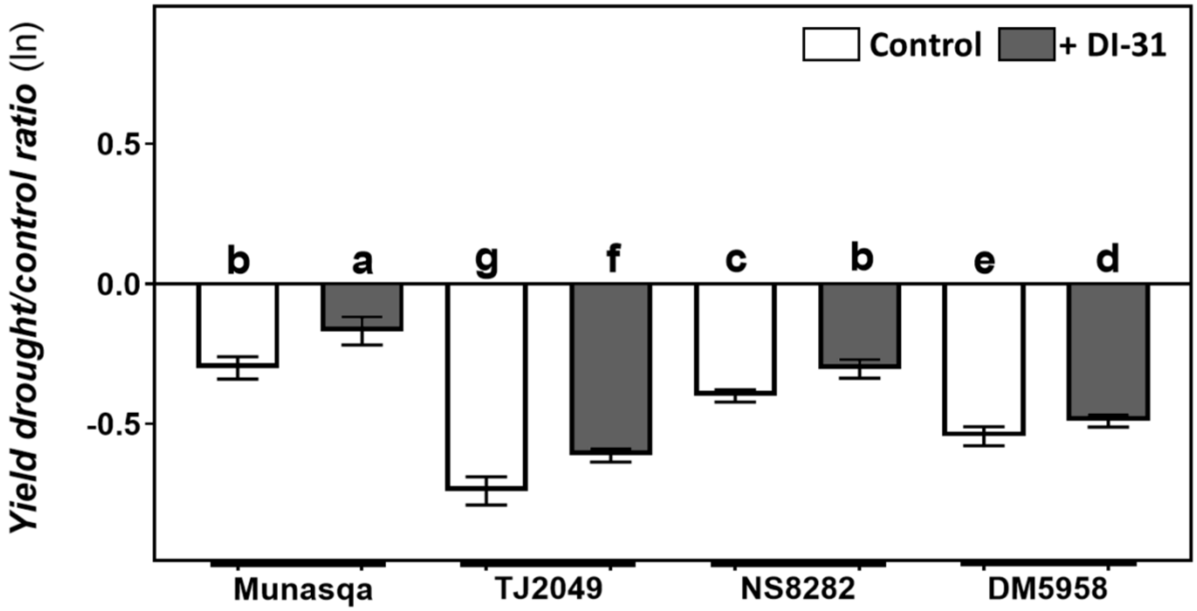

Fig. S2 Effect of DI-31 (2.23 µM) applications in soybean yield drought/control ratio. The cultivars Munasqa, TJ2049, NS8282 and DM5958 were sprayed with DI-31 every 21 days throughout the plant cycle and submitted to well-watered (Ψs = -0.05 MPa) and drought (Ψs= -0.65 MPa) conditions. Ratios were calculated using the data from control and R_5 stage treatments. Colour bars indicate control plants (white) or DI-31 treated ones (grey). Data are presented in natural logarithm (ln) means of the ratio stressed/control ± SE of an independent experiment (n=480). Different letters indicate significant differences ($P \leq 0.05$) ANOVA with *post hoc* contrasts by Tukey's test.
