# Supplementary material for: A brassinosteroid functional analogue increases soybean drought resilience
Source: Sci Rep. 2022 Jul 4;12:11294. doi: 10.1038/s41598-022-15284-6 (PMC9253120; doi:10.1038/s41598-022-15284-6)
Supplement: Supplementary file 3 — Supplementary Information 3. [file 41598_2022_15284_MOESM3_ESM.pdf]

**Manuscript:** A brassinosteroid functional analogue increases soybean drought resilience

**Supplementary File 1**

## Methods

Data corresponding to the "*DI-31 effect in soybean physiology under drought*" experiments were submitted to Principal Component Analysis (PCA). To determine which parameters best explained the phenotypic variability observed, a first PCA was performed using 28 parameters data. Subsequently, data were grouped by biological processes in (**i**) "Photosynthesis, Growth and Water relations", (**ii**) "Stress response", and (**iii**) "Nodulation and Nitrogen homeostasis" sets and submitted to individual PCA analysis. Finally, mean values of the 28 parameters evaluated were normalised and graphed in a double gradient heatmap (Fig. **II**) (GraphPad software 8.4.3). Data were normalised in fractions from 0 to 1, where 0 and 1 were considered the smallest and largest values for each data set, respectively.

## Results

To determine which markers best explained the phenotypic variability observed in well-watered and drought-stressed plants treated with DW or DI-31, a first PCA analysis was performed for all the 28 parameters evaluated in Munasqa plants (Fig. I**a**). Here, the first two principal components (PC) explained 83.7% of total variation (PC1 = 58.9% and PC2 = 24.8%). In PC1, the data were separated by the water availability treatments, and the "drought" plot (coloured in green) showed the biggest discrimination. PC1 revealed a positive association of LWR, CTD, chlorophyll and ureide content with the "control" plot (coloured in blue). In contrast, POX, CAT, sugars and nitrate content were associated with the "drought" plot. At the same time, in PC2, the data were separated by DW and DI-31 application, and the "drought + DI-31" plot (coloured in grey) exhibited the greatest discrimination. A positive association was found between foliar area, WUE, ureide relative abundance and biological N fixed with the "control + DI-31" plot (coloured in yellow). Meanwhile, the SOD, APX, carotenoids, and proline were related to the "drought + DI-31" plot. Compared to the previous one, the individual PCAs performed for each data set showed similar associations among parameters and DI-31. In the PCA corresponding to the "Photosynthesis, Growth and Water relations" set (Fig. I**b**), the first two PC explained the 87.3% of total variation (PC1 = 56.6% and PC2 = 30.7%). Here, the "drought" plot showed the biggest discrimination, but the data distribution didn't correspond to a particular type of treatment. A clear association was observed between CTD and the "control" plot, while foliar area, RWC and WUE were more related to the "control + DI-31" one. No associations were found between parameters and the "drought" plot, while indicators such as $PI_{abs}$, LAR and SLA were related with the "drought + DI-31" plot. Regarding the "Stress response" PCA (Fig. I**c**), the first two PC explained the 85.8% of total variation (PC1 = 59.6% and PC2 = 26.2%). In PC1, data was separated by water availability treatments, and the "control" plot showed the biggest discrimination. Here, chlorophyll was linked to the "control" plot, FRAP, CAT and POX were associated with the "drought" plot, and SOD, APX, carotenoids and proline were again related to the "drought + DI-31" plot. In PC2, the "drought" and "drought + DI-31" plots showed the biggest discrimination, yet, the data couldn't be grouped based on a particular type of treatment. Finally, in the "Nodulation and Nitrogen homeostasis" PCA (Fig. I**d**), the first two PC explained the 98.1% of total variation (PC1 = 82.6% and PC2 = 15.5%). In PC1, data were separated by water availability treatment, and the "drought" plot showed the biggest discrimination. Here, the ureide content was associated with the "control + DI-31" plot, while the active nodules parameter

was more related to the "control" plot. In addition, the *in vivo* NR activity and the nitrate content were associated with the "drought" plot. In PC2, only the "control" and "control + DI-31" plots exhibited some discrimination.

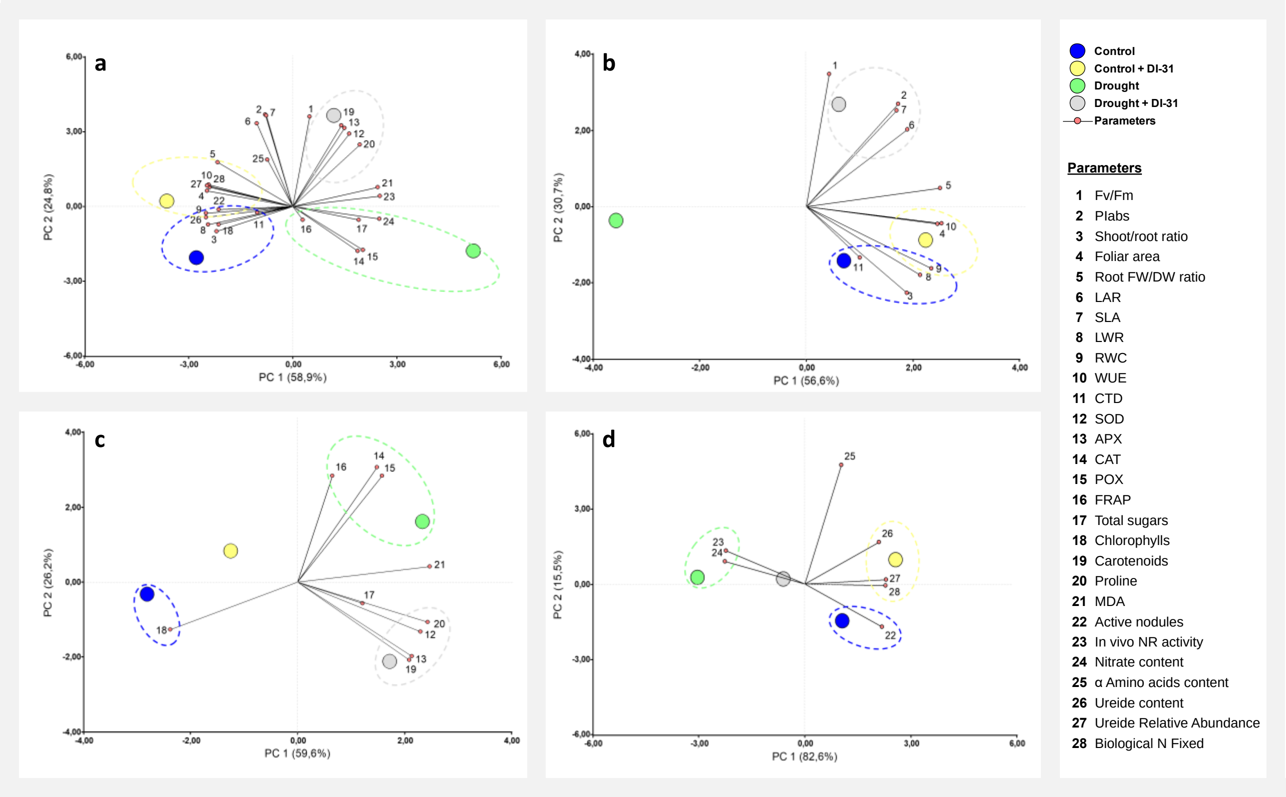


**Fig. I** PCA for determining parameters interaction with treatments. A first PCA was performed using (**a**) all 28 parameters; then data were reanalysed by groups in (**b**) "Photosynthesis, Growth and Water relations" set, (**c**) "Stress response" set, and (**d**) "Nodulation and Nitrogen homeostasis" set. All PCAs were performed using the data from *cv* Munasqa plants submitted to well-watered (Ψs = -0.05 MPa) and drought (Ψs= -0.65 MPa) conditions for ten days.

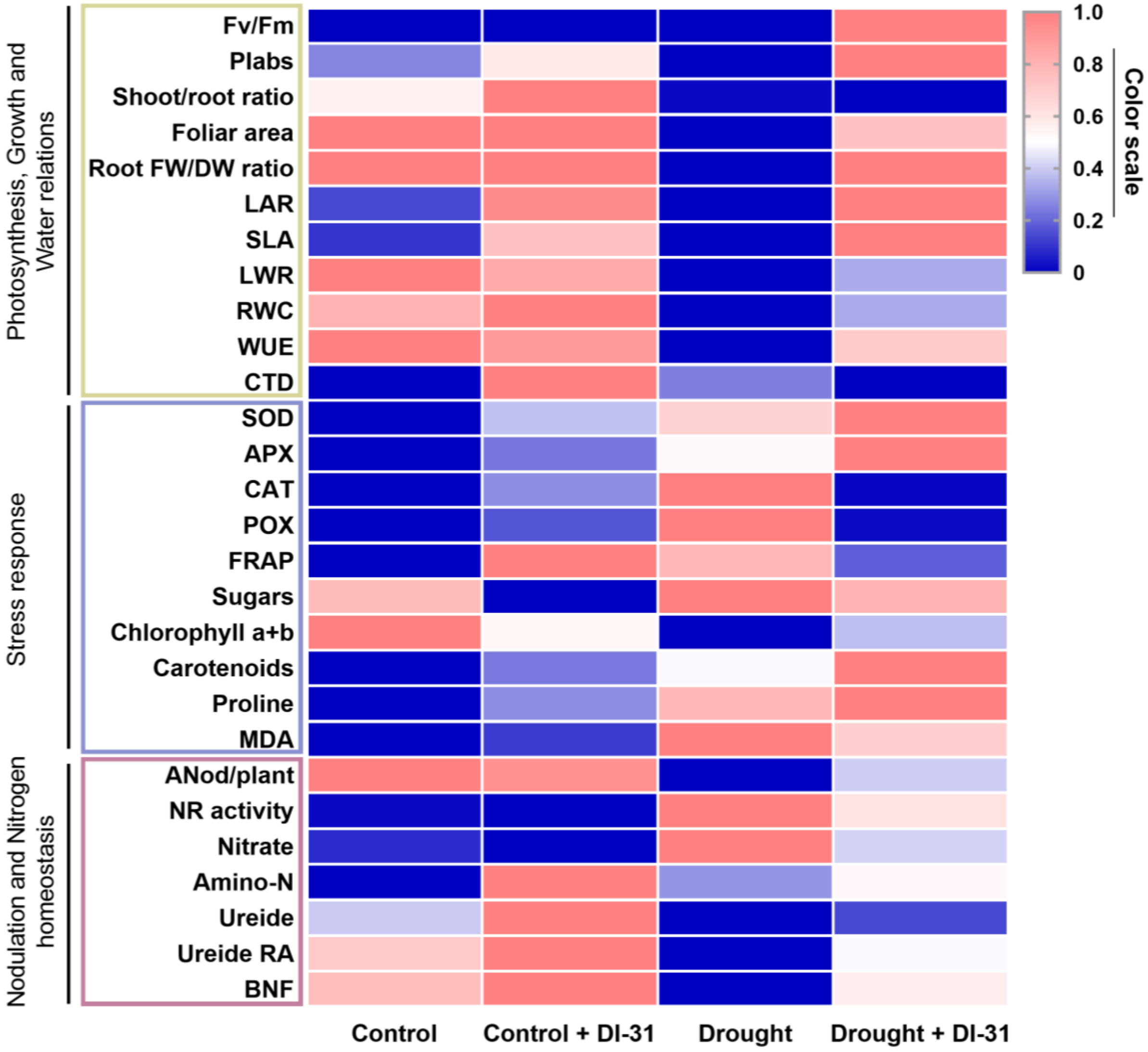


**Fig. II** Double gradient heatmap of DI-31 (2.23 µM) effect in soybean photosynthesis, growth, water relations, stress response, nodulation and nitrogen homeostasis parameters. Data from the 28 parameters were normalised in fractions from 0 to 1, where 0 and 1 were considered the smallest (blue) and largest (orange) values for each data set, respectively.
